# Understanding the Natural and Socioeconomic Factors behind Regional Longevity in Guangxi, China: Is the Centenarian Ratio a Good Enough Indicator for Assessing the Longevity Phenomenon?

**DOI:** 10.3390/ijerph15050938

**Published:** 2018-05-08

**Authors:** Qucheng Deng, Yongping Wei, Yan Zhao, Xuerong Han, Juan Yin

**Affiliations:** 1 School of Earth and Environmental Sciences, The University of Queensland, Brisbane 4067, Australia; q.deng@uq.edu.au (Q.D.); yanzhao.cas@gmail.com (Y.Z.); 2 Key Laboratory of Environmental Change and Resources, Guangxi Teachers Education University, The Ministry of Education, Nanning 530001, China; 3 Guangxi Zhuang Autonomous Region Environmental Monitoring Center, Nanning 530028, China; amelly@yeah.net; 4 Department of Management Science and Engineering, Guangxi University of Finance and Economics, Nanning 530003, Guangxi, China; yinjuan101@163.com

**Keywords:** regional longevity, natural and socioeconomic indicators, spatial analysis, geographically weighted regression, Guangxi

## Abstract

Despite a number of longevity indicators having been used in previous longevity studies, few studies have critically evaluated whether these indicators are suitable to assess the regional longevity level. In addition, an increasing number of studies have attempted to determine the influence of socioeconomic and natural factors on regional longevity, but only certain factors were considered. This study aims to bridge this gap by determining the relationship between the 7 longevity indicators and selecting 24 natural and socioeconomic indicators in 109 selected counties and urban districts in Guangxi, China. This study has applied spatial analysis and geographically weighted regression as the main research methods. The seven longevity indicators here refer to centenarian ratio, longevity index, longevity level, aging tendency, 80^+^ ratio, 90^+^ ratio, and 95^+^ ratio. Natural indicators in this study mainly refer to atmospheric pressure, temperature, difference in temperature, humidity, rainfall, radiation, water vapor, and altitude. Socioeconomic indicators can be categorized into those related to economic status, education, local infrastructure, and health care facilities. The results show that natural factors such as the difference in temperature and altitude, along with socioeconomic factors such as GDP, might be the most significant contributors to the longevity of people aged 60–90 years in Guangxi. The longevity index and longevity level are useful supplementary indexes to the centenarian ratio for assessing the regional longevity.

## 1. Introduction

Aging is a challenge for the global community due to its significant socioeconomic implications. It is well known that the elderly population is more vulnerable due to its weaker immune system and hypofunction, and its life expectancy may be influenced by many factors including genetic, medical, natural, and economic aspects [1,2,3,4,5]. China has become an aging society where the proportion of the population aged 65 years and older reached 8.87% in 2010 and will comprise approximately 25% of the total population by 2050 [6,7]. Thus, China, as the developing country with the largest elderly population, will experience dramatic changes in age structure and subsequent impacts exceeding those of any other country. It is, therefore, important to understand how natural and socioeconomic factors influence regional longevity in China for the development of a healthy aging society.

There are no agreed indicators of longevity in the current literature. Centenarian prevalence has been regarded as one of the most important indicators by which to measure longevity [8,9,10]. Centenarian prevalence refers to the number of centenarians per 100,000 people and could directly reflect the extreme longevity of the total population in a region and the percentage of aged population in a way that makes it easy to draw comparisons between regions. For example, it has been the principal longevity indicator among studies in Italy as a whole and many regions in China [3,10,11,12]. However, the centenarian prevalence could be influenced by local birth rate, migration, and the proportion of local extreme elderly populations [10]. For example, as the total number of centenarians is very limited, a small number of villages with a high elderly population or families with a large number of aged family members in a region could dramatically increase the centenarian prevalence in the area. Other longevity indicators include: the longevity index (the proportion of the population above 90 years old over the aging population, 90^+^/65^+^), longevity level (the proportion of the population above 80 years old over the aging population, 80^+^/60^+^), aging tendency (the proportion of the aging population over the total population, 60^+^/total population), and the proportion of the elderly population (80^+^ ratio, 90^+^ ratio, 95^+^ ratio) within the total population—all of which have been widely used to measure regional longevity. The longevity level and longevity index can reflect the relatively old and eldest people in the older population base and reduce the impact of the age structure to some extent, but they still cannot totally reduce the impact of the age structure. The aging tendency can also be used to help evaluate the overall local elderly proportion in the population; however, this indicator does not help to measure the extreme local longevity ratio among the total population. Moreover, the proportions of 80^+^ ratio, 90^+^ ratio, and 95^+^ ratio could be used to reflect the proportion of these extremely elderly groups within the total population but do not provide a comprehensive view of the total elderly population. Therefore, further research is needed on selection and use of these longevity indicators.

It is well argued that regional longevity is influenced by a combination of multiple socioeconomic and natural indicators. The term “natural indicators” refers to those indicators in the natural world that could impact human health, such as climate factors [2], air quality [12,13,14,15], water quality [16,17], and micronutrient distribution in soil [18]. Similarly, socioeconomic indicators are usually extracted from aspects of the economic index [1,5,8], local infrastructure [19], energy consumption [5], and industrialization [20] that could indirectly influence people’s life expectancy. The majority of regional longevity studies tend to focus on exploring the association between single longevity indicators and a few selected socioeconomic and natural factors. For example, Liu et al. examined the effect of soil trace elements on longevity in populations across the provinces in China and identified several soil trace elements were the most influential [18]. Liu et al. examined the association between the chemical characteristics of natural water and regional longevity in Xinjiang and identified the beneficial trace elements to human health [17]. From the socioeconomic prospective, Kim and Kim examined income level, urban population, mean year of schooling, and Internet access to examine how socioeconomic development could benefit regional longevity [1]. Many recent studies have examined the associations between regional longevity with several selected principal factors. Therefore, while these studies have contributed to understanding regional longevity, the key findings derived from them may not reflect the reality of regional longevity, which is a system consisting of many factors. Thus, these studies may have limited implications for the development of a healthy aging society.

This study aims to investigate the relationship between regional longevity and both natural and socioeconomic factors. For this purpose, 109 counties and urban districts in Guangxi, 7 longevity indicators, and 24 natural and socioeconomic factors were chosen. It is expected that the findings of this study will lead to a better understanding of the consistency of regional longevity indicators as well as a more comprehensive understanding of the socioeconomic and natural factors that influence regional longevity in order to promote the development of a healthy aging society.

## 2. Methods

### 2.1. Case Study Description

**Figure 1:**
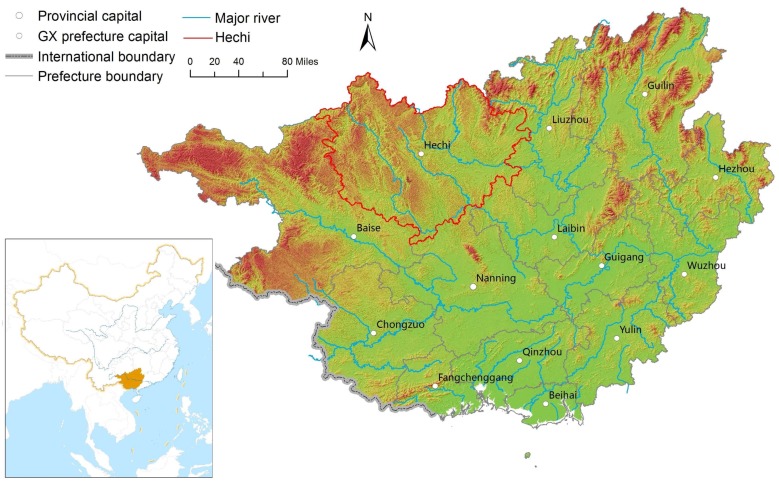
Case study area—Guangxi.

**Figure 2:**
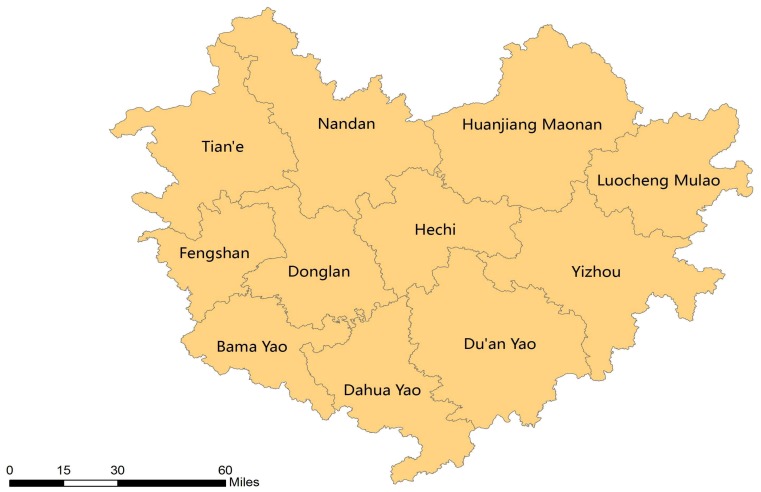
Eleven counties in Hechi city in Guangxi, China.

Guangxi is situated in southern China in a subtropical climate with a variety of topographical features across the province (Figure 1). It includes 109 counties and urban districts [21]. Most of the plain and basin areas in Guangxi are located in the middle and southern area, with mountains and rolling-hilly areas located in the northeastern and northwestern area. Guangxi is a less economically developed area, with a lower economic scale compared to the average economic level in China [22]. Moreover, there is uneven development of the economy and industrialization within the province [19]. Guangxi has a population of approximately 51 million and maintains relatively high proportions of long-lived people according to various National Population Censuses [23]. One of its cities, Hechi, has been recognized by the International Expert Committee on Population Aging and Longevity as the first longevity city in China in 2016 for its high centenarian ratio (17.9/100,000) [24]. Also, six of Hechi’s counties (Bama, Donglan, Fengshan, Yizhou, Dahua, and Tian’e) have been noted as high-longevity counties due to their having leading centenarian ratios among the counties in China (Figure 2) [24]. With a substantial elderly population base, varied subtropical topography, a mild climate, and significant economic and educational differences across the province, Guangxi is an ideal region for examining how regional longevity is associated with multiple natural and socioeconomic factors.

### 2.2. Defining the Old-Age Population

As discussed earlier, no single indicator is good enough to assess regional longevity. Seven longevity indicators (centenarian ratio, longevity index, longevity level, aging tendency, 80^+^ ratio, 90^+^ ratio, and 95^+^ ratio) were chosen to comprehensively reflect the regional extreme longevity, the longevity of the elderly within the population, and the overall elderly population in Guangxi in this study. These indicators, their rationale, and studies in which these indicators were applied are summarized in Table 1.

It should be noted that the elderly population is defined as over 65 years internationally but over 60 in China, and that both standards have been applied according to the different definitions of longevity indicators [25].

### 2.3. Choosing the Factors Influencing the Old-Age Structure

Both natural factors and socioeconomic factors were selected to understand their influence on regional longevity. As discussed earlier, to reflect the reality of regional longevity, a range of natural, social, and economic factors as broad as possible were included. More specifically, the selection of these factors was based on relevant literature reviews and data availability. The selected natural indicators mainly included climate-related aspects regarding atmospheric pressure, temperature, difference in temperature, humidity, rainfall, radiation, and water vapor and altitude. 

The socioeconomic indicators were divided into the following principal sections: economic indicators, education indicators, local infrastructure, and health care facilities. From the economic perspective, seven economic indicators were selected to represent different economic development levels across areas in Guangxi. Notably: primary industry, secondary industry, tertiary industry, and government revenue were chosen to represent the comprehensive regional economic development. GDP per capita was also selected, as it is an indicator that reflects citizens’ financial capacity, which could have positive impacts on improving life expectancy, as the condition of being wealthy enables people to have better education and consume more medical services [19].

For the education aspect, the selected education indicators included the numbers of primary schools secondary schools, population with primary school education and secondary school education. These factors could reflect the local education capacity and general education level. The local infrastructure indicators regarding the number of mobile telephone subscribers and annual electricity consumption were selected to reflect the telecommunication conditions and electricity access and consumption in the area. Health care facilities in the townships, including hospitals, were chosen because they could reflect how convenient it is for local residents to receive medical care. The denser the distribution of hospitals in the area, the more access local people would have to medical treatment and advice to improve the quality of their health [19]. The number of hospital beds was also selected, as this indicator could reflect the capacity of regional medical treatment facilities. These selected natural and socioeconomic indicators are summarized in Table 2.

### 2.4. Data Sources and Analytical Methods

#### 2.4.1. Data Sources

Population data was collected from the demographic database of the Sixth National Population Census of China of 2010 [21]. Longevity indicators were generated from the collected population data and used as the dependent variables in the study. The climate and socioeconomic indicators, which were independent variables, were collected from China’s Meteorological Data-Sharing Service System, the demographic database of the Sixth National Population Census of China, 2010, and the Guangxi Statistical Yearbook, respectively [21,23,27]. Elevations of Guangxi were extract from the 1:50,000 scale map. The quality of censuses in China has been criticized in the literature [28], however, there are no better data sources for such comprehensive data required in this study. In addition, most scholars agree that the quality of population censuses in China has risen after 1982 as the United Nations Population Fund provided support and the Chinese Government introduced rules to control the quality of the survey data and especially for age verification. Scholars agree that the Sixth National Population Census of China is of reasonably good quality [29,30].

#### 2.4.2. Statistical Analysis

This study has used Excel 2016 (Microsoft, Redmond, WA, USA) to calculate the longevity indicators from the Sixth National Population Census of China, 2010. The correlation between the seven longevity indicators was made with the SPSS 22.0 (IBM, New York, NY, USA). The ArcGIS 10.40 (ESRI, Redlands, CA, USA) was used to generate the spatial distribution of regional longevity and selected natural and socioeconomic indicators. Most importantly, this study applied spatial autocorrelation analysis to detect the autocorrelation among independent variables. A geographically weighted regression model was then used to determine the relationship between the dependent variables and independent variables, which can effectively reduce the negative impact of autocorrelation. The formula of the spatial autocorrelation analysis and geographically weighted regression and the detail process of the experiment are introduced in the following sections.

#### 2.4.3. Spatial Autocorrelation Analysis

The Moran index (Moran’s *I*) was used to measure spatial autocorrelation of indicators. Moran’s *I* can be presented by the following formula [31]:

(1) $$I=\frac{n\sum_{i=1}^{n}\sum_{j=1}^{n}w_{ij}(x_{i}-\bar{x})(x_{j}-\bar{x})}{S_{0}\left(\sum_{i=1}^{n}\sum_{j=1}^{n}w_{ij}\right)\sum_{i=1}^{n}{\left(x_{i}-\bar{x}\right)}^{2}}$$

where *n* is the number of spatial units indexed by *i* and *j*; *x* is the variable of interest; *x_i_* and *x_j_* are the values of the observed variable at sites *i* and *j*; $\bar{x}$ is the mean of *x*; and the weights *W_ij_* are written in a (*n* × *n*) weight matrix. The weight matrix depicts the relationship between an element and its surrounding elements. Weight can be based on contiguity relationship or distance. The value of Moran’s *I* usually ranges from −1 to +1. The positive values of Moran’s *I* indicate positive spatial autocorrelation; in contrast, the negative values of Moran’s *I* indicate negative spatial autocorrelation.

With regard to the statistical hypothesis testing, values of Moran’s *I* could be tested based on their Z-scores; for instance, in the situation of |Z| > 1.96, spatial autocorrelation is significant at the 0.05 confidence level, and when |Z| > 2.58, this suggests that spatial autocorrelation is significant at the 0.01 confidence level.

In this study, Spatial Autocorrelation Analysis (Moran’s *I*) was first employed by ArcGIS 10.40 (ESRI, Redlands, CA, USA) to determine the relationship for the 24 independent variables. The results showed that most of the variables have relatively higher autocorrelations, for example, the independent variables regarding the atmospheric pressure, difference in temperature, humidity, rainfall, radiation, temperature, water vapor, number of primary schools, and number of secondary schools. Moran’s *I* ranged from 0.44 to 0.94, and these independent variables had relatively positive Z and P values. Thus, the conventional regression analysis might not the best option in this study to determine the relationship between the independent and dependent variables. Hence, we chose the geographically weighted regression, which could effectively reduce the impact of spatial autocorrelation.

#### 2.4.4. Geographically Weighted Regression (GWR)

We used GWR with the software GeoDa 1.10 (Chicago, IL, USA) to determine the relationship between the independent variables regarding socioeconomic factors, natural factors, and dependent variables with regard to longevity indicators. The geographically weighted regression model can be presented by the following formula (2):

(2) $$y_{i}=\beta_{0}(u_{i},v_{i})+\sum_{k}\beta_{k}(u_{i},v_{i})x_{ik}+\varepsilon_{i}$$

where *y_i_* refers to the dependent variables such as centenarian ratio, longevity index, longevity level, aging tendency, 80^+^ ratio, 90^+^ ratio, and 95^+^ ratio at location *i*. (*u_i_*, *v_i_*) means the coordinates of the centroid at location *i*. *β*o(*u_i_*, *v_i_*) refers to the intercept for location *i*. *β*k(*u_i_*, *v_i_*) means the local parameter for independent variable k at location *i*. *x_ik_* is the value of independent variable *k* at location *i*. *εi* is the error term for location *i*.

(3) $$\beta (u_{i},v_{i})={\left(X^{T}W(u_{i},v_{i})X\right)}^{-1}X^{T}W(u_{i},v_{i})Y$$

where *β* (*u_i_*, *v_i_*) means the local regression coefficient at location *i*. *X* refers to the matrix of the independent variables of the socioeconomic and natural factors. *Y* is the vector of the dependent variable of longevity indicators [32].

In the Geographically Weighted Regression section, we first focused on univariate analysis to determine the most significant independent variables that have contributed to the dependent variables in a situation where many independent variables were entered into the equation. In this model, no autocorrelation or multicollinearity problem existed, as each variable was entered one by one. In the Multivariate Analysis section, we first included the selected independent variables, assigned a Rook contiguity weights file, and conducted conventional least squares, spatial-lagged regression and spatial error to determine the relationship. As the ordinary least squares showed strong autocorrelation, a spatial error model and a spatial lag model were used to eliminate the influence of autocorrelation. The multicollinearity condition number is less than 100 for each model in this study, suggesting that there is no serious multicollinearity problem.

## 3. Results

### 3.1. The Spatial Distribution of Different Elderly Age Groups in Guangxi

**Figure 3:**
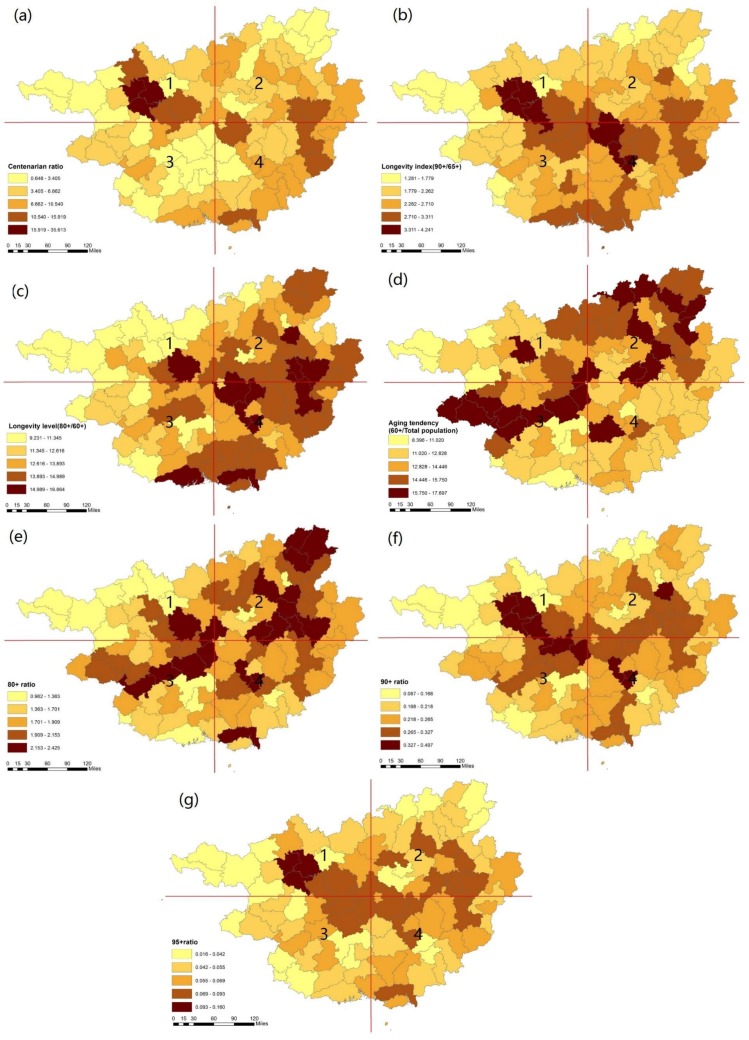
Spatial distribution of seven longevity indicators in Guangxi (**a**–**g**).

There are clear regional differences among the seven longevity indicators across the study areas (Figure 3). Figure 3a shows that the centenarian ratio varies across the region. The counties with the highest centenarian ratio are located in the northwestern area: Bama Yao ranks first (36 per million inhabitants), followed by Donglan (31 per million inhabitants) and Fengshan (28 per million inhabitants). These counties are all located inside the middle of the 1st quadrant. On the other hand, the regions with a lower centenarian ratio are distributed in the southwestern area of Nanning and the western area of Baise in the 3rd and 1st quadrants. The centenarian ratio tends to be highly concentrated. The Bama Yao, Donglan, and Fengshan counties that are located in the southwestern area of Hechi have been recognized as one of the most famous longevity regions in the world.

Figure 3b illustrates the distribution of the longevity index, in which the northwestern and central areas have a higher longevity index. The Bama Yao (4.24), Donglan (4.11), Dahua Yao (3.58), and Fengshan (3.41) counties in Hechi city have the leading longevity index and are located in the middle of the 1st quadrant. In contrast, the western area of Baise city has a relatively low longevity index. Similarly, the 90^+^ ratio in Figure 3f and 95^+^ in Figure 3g show a similar longevity trend, which is higher in the northwestern area. Similarly, the higher values of both the centenarian ratio and longevity index, 90^+^ and 95^+^, all tended to be located in the northwestern area of Guangxi inside the 1st quadrant.

Figure 3c shows the longevity level in the study area. The areas located in the 2nd and 4th quadrants have high longevity levels, while the areas in the western area in the 1st and 3rd quadrants have lower levels. The 80^+^ ratio and aging tendency seem to have similar distributions in Figure 3e and Figure 3d. The high values of the 80^+^ and aging tendency ratios are distributed in the northeastern areas in the 2nd quadrant and partial areas in southwestern areas in the 3rd quadrant. The aging tendency tends to be distributed more widely in Guangxi than the 80^+^ ratio, which presents the wide distribution of the younger elderly population.

### 3.2. The Spatial Pattern of Natural and Socioeconomic Factors That Might Impact Longevity Distribution

**Figure 4:**
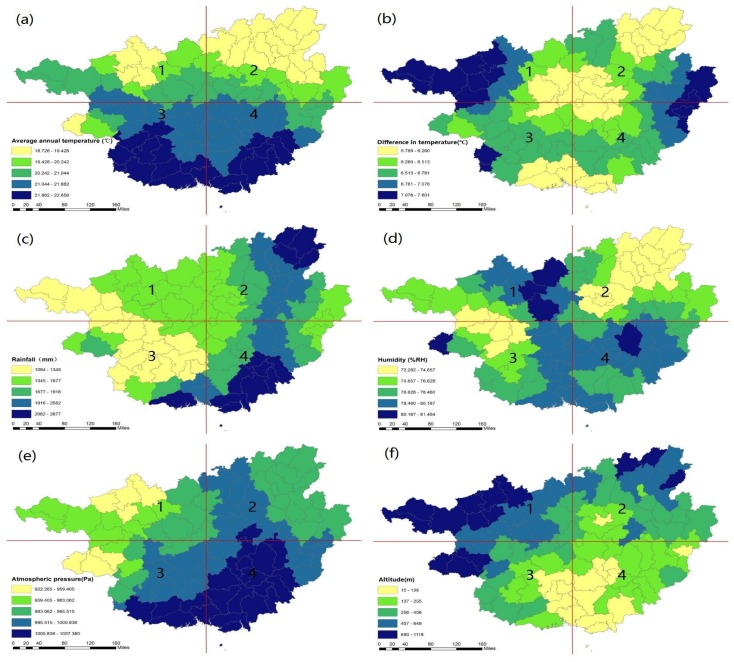
The spatial distribution of climate indicators in Guangxi (**a**–**f**).

**Figure 5:**
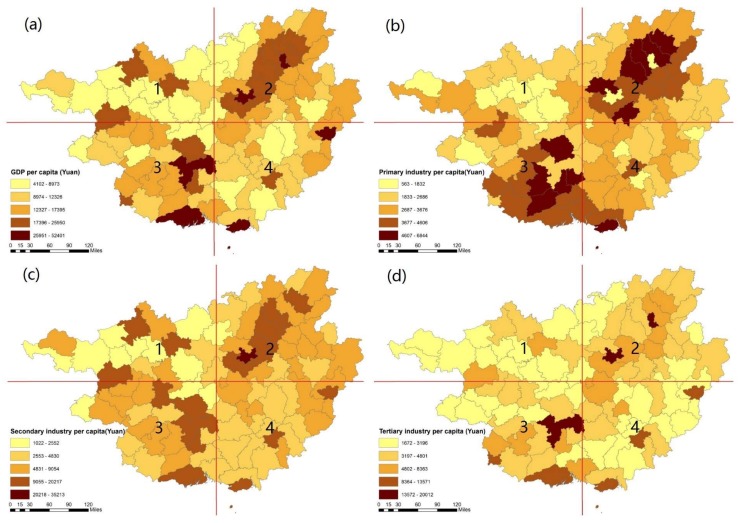
The spatial distribution of economic indicators in Guangxi (**a**–**d**).

**Figure 6:**
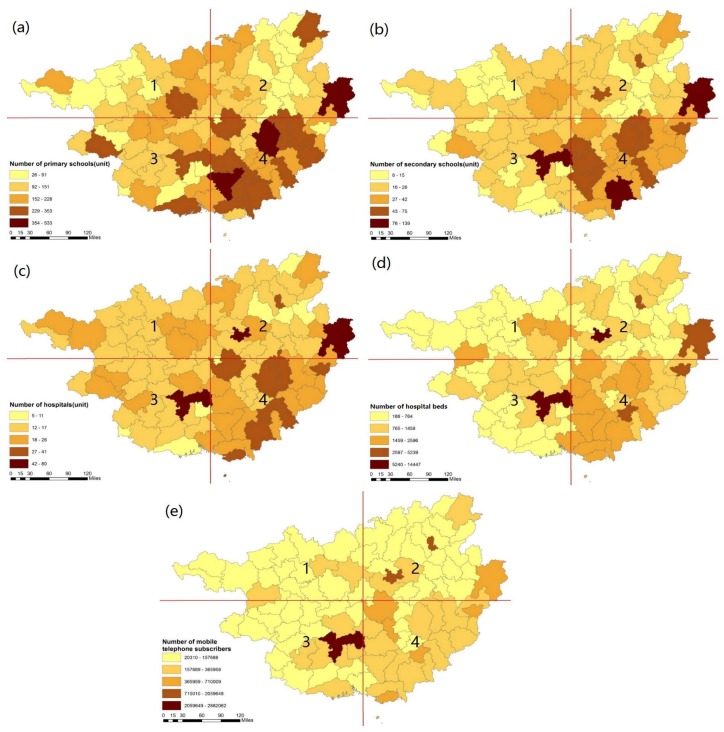
The spatial distribution of educational, infrastructural, and medical care indicators in Guangxi (**a**–**e**).

The spatial distribution of the selected climate, economic, and education indicators that may impact the regional longevity distribution in Guangxi is shown in Figure 4, Figure 5 and Figure 6, respectively. Figure 4a shows that Guangxi’s average annual temperature gradually decreases from south to north, with a peak temperature of approximately 23 °C in the 3rd and 4th quadrants and a low of around 18 °C in the 2nd quadrant. Figure 4b illustrates the difference in temperature in Guangxi, with the highest difference of 7 °C located at the west and east sides of Guangxi in the 1st and 2nd quadrant and a moderate temperature difference of approximately 6 °C in the middle area. Figure 4c presents the annual precipitation throughout Guangxi, with a high concentration across the northeast area and southwest area of around 2300 mm in the northwestern area in the 2nd quadrant and the southeastern area in the 4th quadrant. The middle areas have temperate precipitation, while the western area has light precipitation of roughly 1000 mm. Figure 4d shows the various humidity conditions across the province of Guangxi. The northern area has the highest humidity conditions in the 1st quadrant, while the southeast section also has high humidity in the 4th quadrant. Meanwhile, the western and northeastern areas of Guangxi have lower humidity levels. Figure 4f shows the average altitude in Guangxi. The highest areas are in the northwestern areas of Guangxi. Compared to the longevity indicators in Figure 1, most of the longevity areas in Guangxi tend to be located in the areas where the temperature and temperature difference is moderate, humidity is high, and altitude ranged from 400–700 m.

Figure 5 shows the significant differences in economic indicators across Guangxi. The indicators of primary industry per capita, secondary industry per capita, tertiary industry per capita, and GDP per capita show that economic activity is clustered around the southwestern and northeastern areas. These areas belong to the major cities of Guangxi, namely Nanning, Liuzhou, and Guilin. The urban districts in Guangxi are mainly located in the high value area. However, the centenarian concentrated areas of Bama Yao, Fengshan, and Donglan in the 1st quadrant have weaker economic activity. The higher value for the longevity index in the 90^+^ and 95^+^ areas seems to be different from those in the economically developed areas. The high value of the 80^+^ and aging tendency indicators tend to be located similarly in relation to the high value areas of economic indicators and are distributed mainly in the northeastern and southwestern areas in the 2nd and 3rd quadrants.

Figure 6 shows the spatial pattern of education indicators and infrastructure and telecommunication indicators in Guangxi. Numbers of primary schools (Figure 6a), secondary schools (Figure 6b), hospitals (Figure 6c), beds in hospitals (Figure 6d), and mobile subscribers (Figure 6e) have high concentrations in the southeastern area in Guangxi, while the northeastern and southwestern areas have lower concentrations. The municipal districts of Nanning, Liuzhou, and Guilin have relatively higher education and infrastructure indicators compared to other areas in Guangxi. The Bama Yao, Donglan, and Fengshan counties, with the highest centenarian ratio in Hechi city, are areas where the education and medical infrastructure levels are relatively lower.

### 3.3. The Statistical Relationship between the Seven Longevity Indicators and Selected Natural and Socioeconomic Indicators

Table 3 and Table 4 show the results of univariate and multivariate analyses, respectively, based on geographically weighted regression analysis. Based on the univariate analysis, there are many natural and socioeconomic factors correlating the aging tendency, 80^+^ ratio, and longevity level. For example, aging tendency in this study tended to be associated with difference in temperature, number of secondary schools, number of hospital beds, GDP, tertiary industry, and population with primary school education. The 80^+^ ratio tended to be associated with difference in temperature, GDP, altitude, and rainfall. The longevity level tended to be negatively associated with difference in temperature and altitude, and positively associated with the other selected natural and socioeconomic factors, but coefficients in both cases were relatively low. Based on the multivariate analysis, the aging tendency was related with difference in temperature and tertiary industry. The 80^+^ ratio shows the associated factor related difference in temperature. Regarding the longevity level, difference in temperature and altitude were relatively significant. 

Regarding the longevity index, the univariate analysis shows an association between the difference in temperature and altitude; the multivariate analysis also shows that the longevity index is associated with difference in temperature. The centenarian ratio and 95^+^ ratio have not entered the equation with any of the selected factors in both the univariate and multivariate analyses.

### 3.4. Correlation between the Seven Longevity Indicators

Table 5 shows the results of the correlation analysis of the seven longevity indicators in Guangxi. The centenarian ratio has a strong correlation with the 95^+^ ratios and longevity index. The longevity index seems to have a strong correlation with the 95^+^ ratio, centenarian ratio, longevity level and 90^+^ ratio. Regarding the longevity level, it has a correlation with the 90^+^ ratio, 80^+^ ratio, and a strong correlation with the longevity index and 95^+^ ratio.

## 4. Discussion

This study aimed to examine regional longevity on a provincial scale in Guangxi by investigating the relationship between 7 longevity indicators and 24 natural and socioeconomic indicators that cover climate, economic, educational, infrastructural, and health care facility aspects. This study has methodically applied ArcGIS 10.40 in order to analyze the differences among the major indicators from a spatial perspective, and also used geographically weighted regression to investigate the association between the longevity indicators and the natural and socioeconomic indicators. The key findings of this study and the implications for future research direction and longevity management are summarized below.

Based on univariate and multivariate analyses conducted by the geographically weighted regression model, two important findings can be drawn from this study. First, there were no statistically significant variables influencing the centenarian ratio and 95^+^ ratio in both the univariate analysis and multivariate analysis. Second, mild climate and socioeconomic conditions in Guangxi may also be significant factors that have contributed to the health conditions and relative proportion of the population of 60- to 90-year-olds at the provincial level.

The second finding has shown similar conclusions that elderly people are likely to be distributed in places where the environment and climatic condition are better [2,33]. In addition, there are studies that argue that better economic conditions can help maintain a relatively high proportion of old-age populations [6,19]. The reason provided was that economically developed areas could provide better medical services, a complete education system, and a social security system, which could significantly promote life expectancy and achieve better regional longevity [5,6,19]. Our findings suggested that mild climate traits as well as socioeconomic factors are both important for the longevity phenomenon in Guangxi. On the other hand, for longevity level, aging tendency, 80^+^ ratio, and longevity index indicators, the geographically weighted regression showed that the difference in temperature is negatively correlated with all the four longevity indicators. These results might indicate that dramatic change in temperature is an important driver that reduces the longevity phenomenon in Guangxi, as the difference in temperature could increase the incidence of many diseases in the elderly population. 

The extreme longevity indicators regarding the centenarian ratio and 95^+^ ratio seem to show no correlation with either the natural or socioeconomic factors. These results might suggest that the extreme elderly aged over 95 years in Guangxi might be an accidental phenomenon based on the selected indicators in this study. Some longevity studies in China have concluded that very elderly people (in particular, those aged over 90 years or centenarians) tend not to rely very much on local economic developmental levels [34], but instead depend on a better natural environment [2]. As a result, these extreme-aged citizens tend to be located in areas where the economy is less developed in locations such as Hainan, Guangxi, and Sichuan [2,11,35]. However, the present study does not show any positive correlation between extreme longevity indicators and the selected climate indicators in Guangxi.

Regarding the longevity indicators, the findings from this study confirmed that no single indicator is good enough on its own to assess regional longevity. In particular, the centenarian ratio, regarded as the most commonly used indicator in regional longevity studies, has no implications as it is not correlated with any selected indicators in this study. The longevity phenomenon is not universal and there are only limited regions that enjoy a high ratio of longevity. The longevity areas in Guangxi have shown uneven distribution, and the distribution of centenarians tends to be denser in the northwestern area. For example, the centenarians in the Guangxi area are mainly located in Bama Yao, Fengshan, and Donglan in Hechi city, which are stable longevity areas in the remote mountainous areas in Guangxi. The genetic and environmental factors might be the most important contributors to the regional longevity in Hechi city [36]. As a result, in Guangxi, the centenarian ratio only reflects the extreme regional longevity in a relatively small area while lacking the correlations between the socioeconomic and natural indicators.

The longevity indicators can measure the longevity from different dimensions. The difference between the centenarian ratio and longevity index is that the centenarian ratio reflects the extremely elderly population out of the total population, and the longevity index reflects the extreme longevity in the elderly population. The centenarian ratio is not correlated with the longevity level, but the longevity index is correlated with the longevity level. This indicates the longevity index could represent the continuity of longevity and could be used as a supplementary index for the centenarian ratio. The longevity level could reflect the secondary increase in the aging population and correlates with the longevity index and 95^+^ population ratio. It also represents the overall level of the longevity phenomenon and longevity continuity. Considering the number of centenarians in the future, it could be used as a useful supplementary index. For example, the city of Hechi has the leading centenarian ratio and longevity index in Guangxi, but its longevity level and 80^+^ ratio/aging tendency only rank in the upper middle among the counties and areas in Guangxi. In this case, only the centenarian ratio might not be able to comprehensively reflect the regional longevity condition in Hechi city. The socioeconomic conditions and development might have an impact on the younger elderly population but do not impact significantly on the extreme-longevity population. As a result, the centenarian ratio, together with indicators such as the longevity index, longevity level, and aging tendency, have helped create a better understanding of regional longevity and reflect the local population structure and are useful supplementary indexes to the centenarian ratio for assessing regional longevity.

Three limitations of this study should be noted. First, this study only focused on a particular year in Guangxi without examining regional longevity in this area over a longer timeframe. Obviously, regional longevity is a relative concept that evolves with time. Second, this study only considered limited natural factors from climate and altitude perspective without taking into account other factors such as landscape condition, drinking water, soil, and air quality, which might be more influential and more closely associated with human health and thus might contribute to longevity in the regions. Third, the concerns of the quality of the statistical data, which might slightly impact the precision of this study, are well-known.

## 5. Conclusions

Regional longevity is a result of both natural and socioeconomic factors. To our knowledge, this is the first study to investigate the relationship between the seven longevity indicators and a number of natural and socioeconomic indicators on a provincial scale. The results indicated that mild climate conditions are more significant contributors to regional longevity than socioeconomic indicators in the case of Guangxi. The factors associated with the extreme elderly (aged above 95 years) in Guangxi are not clear and require further investigation. The longevity index and longevity level are useful supplementary indexes to the centenarian ratio for assessing regional longevity, and a mild and moderate local climate could significantly contribute to improving human health and promoting higher life expectancy.

## Figures and Tables

**Table 1:** Indicators of the old-age structure.

Indicators	Definition	Rationale	References
Centenarian ratio	Number of centenarians per 100,000 people	To reflect the extreme regional longevity rate	Song et al., 2016, [5]
Longevity index	The proportion of 90^+^/65^+^ population	To reflect the extreme longevity among the elderly population	Lv et al., 2011, Song et al., 2016, Magnolfi et al., 2007, [2,5,9]
Longevity level	The proportion of 80^+^/60^+^ population	To reflect the secondary longevity rate of the elderly population	Li et al., 2013, [26]
Aging tendency (60^+^ ratio)	60^+^ elderly population/total population	To reflect the total local elderly population proportion and aging tendency	Wang et al., 2015, [11]
80^+^ ratio	80^+^ elderly population/total population	To reflect the proportion of the second oldest group in the total population	Wang et al., 2016, [6]
90^+^ ratio	90^+^ elderly population/total population	To reflect the proportion of extreme elderly in the total population	Lv et al., 2011, [2]
95^+^ ratio	95^+^ elderly population/total population	To reflect the proportion of extreme elderly in the total population	

**Table 2:** Selected natural and socioeconomic indicators.

Natural indicators	atmospheric pressure ^N^, difference in temperature ^N^, humidity ^N^, rainfall ^N^, radiation ^N^, temperature ^N^, water vapor ^N^, altitude ^N^
Economic indicators	primary industry ^SE^, secondary industry ^SE^, tertiary industry ^SE^, government revenue ^SE^, GDP ^SE^, output of grain ^SE^, urban registration employment rate ^SE^
Education indicators	population with primary school education ^SE^, population with secondary school education ^SE^, number of primary schools ^SE^, number of secondary schools ^SE^
Local infrastructure indicators	number of resident buildings ^SE^, number of mobile telephone subscribers ^SE^, annual electricity consumption ^SE^
Health care facilities	number of hospitals ^SE^, number of hospital beds ^SE^

Note: natural factors: ^N^; socioeconomic factors: ^SE^.

**Table 3:** Results of GWR between seven longevity indicators and selected natural and socioeconomic indicators (univariate analysis).

Longevity Indictors	Univariate Analysis			
	Coefficient	*Z*-Value	Significance	Variables
Centenarian ratio	There are no statistically significant variables			
Longevity Index	−2.326	−1.764	0.078	DT
	−0.005	−2.315	0.021	Altitude
Longevity level	2.94 × 10^−8^	2.906	0.004	PSSE
	4.34 × 10^−4^	3.419	0.001	AP
	−0.009	−2.722	0.007	DT
	−2.40 × 10^−5^	−3.876	0.000	Altitude
	3.12 × 10^−6^	2.057	0.040	NHB
	2.34 × 10^−8^	2.638	0.008	OG
	3.28 × 10^−5^	2.489	0.013	NPS
	2.71 × 10^−8^	3.409	0.001	PI
	2.35 × 10^−5^	2.038	0.042	Radiation
	1.36 × 10^−5^	3.188	0.001	Rainfall
	1.84 × 10^−8^	2.656	0.008	TI
	5.77 × 10^−9^	2.196	0.028	PPSE
	3.12 × 10^−6^	2.092	0.44	GDP
Aging tendency	−2.69 × 10^−8^	−2.440	0.015	TI
	−0.013	−2.523	0.012	DT
	−5.23 × 10^−6^	−2.152	0.031	NHB
	−2.49 × 10^−4^	−1.834	0.067	NSS
	−3.49 × 10^−8^	−1.858	0.063	PPSE
	2.82 × 10^−6^	2.051	0.039	GDP
80^+^ ratio	−0.003	−3.525	0.000	DT
	−2.30 × 10^−6^	−1.738	0.082	Altitude
	2.48 × 10^−6^	2.414	0.016	Rainfall
	2.35 × 10^−5^	2.038	0.042	GDP
90^+^ ratio	−3.99 × 10^−4^	−2.349	0.019	DT
95^+^ ratio	There are no statistically significant variables			

Note: DT: difference in temperature; PSSE: population with secondary school education; AP: atmospheric pressure; NHB: number of hospital beds; OG: output of grain; NPS: number of primary schools; PI: primary industry; TI: tertiary industry; NSS: number of secondary schools; PPSE: population with primary school education.

**Table 4:** Results of GWR between seven longevity indicators and selected natural and socioeconomic indicators (multivariate analysis).

Longevity Indictors	Multivariate Analysis			
	Coefficient	*Z*-Value	Significance	Variables
Centenarian ratio	There are no variables statistically significant			
Longevity Index	−4.426	−1.952	0.051	DT
Longevity level	−1.64 × 10^−5^	−2.407	0.016	Altitude
	−7.71 × 10^−3^	−2.375	0.018	DT
Aging tendency	−0.018	−3.794	0.000	DT
	−3.78 × 10^−8^	−1.989	0.047	TI
80^+^ ratio	−0.002	−2.629	0.009	DT
90^+^ ratio	There were no statistically significant variables			
95^+^ ratio	There were no statistically significant variables			

Note: DT: difference in temperature; TI: tertiary industry.

**Table 5:** The correlation analysis between the seven longevity indicators in Guangxi.

	Centenarian Ratio	Longevity Index	Longevity Level	Aging Tendency	80^+^ Ratio	90^+^ Ratio	95^+^ Ratio
Centenarian ratio	1.000	0.546 **	0.165	0.057	−0.020	0.174	0.571 **
Longevity index	0.546 **	1.000	0.518 **	−0.044	0.083	0.279 **	0.571 **
Longevity level	0.165	0.518 **	1.000	0.154	0.230 *	0.260 *	0.356 **
Aging tendency	0.057	−0.044	0.154	1.000	0.176	0.150	0.213 *
80^+^ ratio	−0.020	0.083	0.230 *	0.176	1.000	0.965 **	0.715 **
90^+^ ratio	0.174	0.279 **	0.260 *	0.150	0.965 **	1.000	0.857 **
95^+^ ratio	0.571 **	0.571 **	0.356 **	0.213 *	0.715 **	0.857 **	1.000

* Correlation is significant at the 0.05 level (2-tailed). ** Correlation is significant at the 0.01 level (2-tailed).
